# Not just mutations: inbreeding depression persists without genetic variation

**DOI:** 10.1093/evlett/qrag008

**Published:** 2026-03-28

**Authors:** Nicolás Bonel, Christoph Grunau, Patrice David

**Affiliations:** Genética y Ecología Evolutiva, CERZOS, CONICET–Universidad Nacional del Sur, Bahía Blanca, Argentina; Centre d’Écologie Fonctionnelle et Évolutive, UMR 5175, CNRS–Université de Montpellier, Université Paul-Valéry Montpellier–École Pratique des Hautes Études–IRD, Montpellier, France; Interactions Hôtes-Pathogènes-Environnements, UMR 5244, Université de Perpignan, CNRS, IFREMER, Perpignan, France; Centre d’Écologie Fonctionnelle et Évolutive, UMR 5175, CNRS–Université de Montpellier, Université Paul-Valéry Montpellier–École Pratique des Hautes Études–IRD, Montpellier, France

**Keywords:** inbreeding depression, *Physa acuta*, epigenetics, inbred lines, self-fertilization, fitness traits

## Abstract

Inbreeding depression (ID)—the reduction in fitness with increasing parental relatedness—is classically attributed to the expression of recessive deleterious mutations in homozygous individuals. Yet, the assumption that ID can only arise from changes in genetic heterozygosity has rarely, if ever, been directly tested. To test this, we produced highly inbred lines (*F* = 0.99999997) of the freshwater snail *Physa acuta* and generated offspring that differed in parental relatedness (self-fertilization, sib or cousin matings) while their parents, produced by crossing two inbred lines, all shared an identical genome. Several fitness traits showed significant declines with increasing parental relatedness. These traits included juvenile survival, body size, and self-fertility, and the magnitude of their decline was equivalent to a substantial fraction of the ID observed in natural, genetically polymorphic populations of *P. acuta*. Individual-based simulations demonstrated that spontaneous mutation rates compatible with natural levels of ID are far too low to account for the magnitude of ID observed here. These findings suggest that non-genetic mechanisms, most plausibly involving heritable epigenetic changes, can generate ID even in genetically uniform populations. This challenges the long-standing view that ID arises exclusively from genetic homozygosity and highlights the need to investigate epigenetic contributions to ID.

## Introduction

Inbreeding depression (ID) is the loss in fitness that occurs in offspring of consanguineous matings and is one of the most general observations in evolutionary biology (Charlesworth & Charlesworth, 1999; Charlesworth & Willis, 2009). Understanding the mechanisms of ID is important because it strongly constrains the evolution of mating systems (self-fertilization rates, self-incompatibility) and is a major concern in plant breeding and animal conservation (Charlesworth & Charlesworth, 1998). ID is classically explained by the presence of recessive deleterious mutations in the genome, whose effects are exposed by homozygosity (Charlesworth & Willis, 2009). Most of the mutations that occur spontaneously at every generation are indeed deleterious and partly recessive (Agrawal & Whitlock, 2011; Charlesworth & Willis, 2009; Huber et al., 2018; Simmons & Crow, 1977). In large diploid organisms, however, genomic mutation rates are typically small, implying that the standing inbreeding load is accumulated through many generations until a steady state of mutation, selection, and drift is reached. This view inspired the idea that a substantial fraction of standing ID could be removed in conditions that drastically reduce heterozygosity by increasing the elimination or fixation rates of alleles.

Genetic purging is expected to occur when systematic inbreeding (such as high selfing rates) exposes deleterious alleles to selection in large populations (Glémin, 2003; Lande & Schemske, 1985). In very small populations, ID can also be lost by drift, as alleles—deleterious or not—become fixed and heterozygosity is reduced (Glémin, 2003; Kirkpatrick & Jarne, 2000). Once homozygosity is attained in a genomic region, the sole mechanism that may reintroduce heterozygosity, and regenerate ID, is the occurrence of spontaneous mutations. Mutations, however, are rare and, within a selfing or sib-mating line, they are quickly lost or fixed, so they are not expected to yield substantial levels of ID. For this reason, highly inbred populations, as well as experimental systems such as recombinant inbred lines (RILs), have often been considered unsuitable for the study of ID, since repeated selfing should purge or fix deleterious recessives and eliminate heterozygosity (Bailey, 1971). In other words, individuals from the same inbred line are essentially genetic clones, and their offspring should show no difference in fitness regardless of whether reproduction occurs through selfing or crossing.

Yet, empirical evidence suggests that ID is difficult to purge completely (Waller, 2021), and residual ID has been documented even after many generations of enforced inbreeding in both natural and experimental populations (Byers & Waller, 1999; Carr & Dudash, 1997; Chelo et al., 2014; Crnokrak & Barrett, 2002). To explain these observations, authors usually refer to factors that theoretically make ID resistant to purge or fixation, such as (i) a highly multilocus architecture of the inbreeding load (many loci of very small effect) that slows down its purging by selection (Charlesworth et al., 1990; Lande et al., 1994), and (ii) physical linkage that can prolong the life of deleterious alleles through pseudo-overdominance, as in the case of linked lethals in repulsion (Waller, 2021; Wang & Hill, 1999; Zhao & Charlesworth, 2016). However, both mechanisms are hard to quantify precisely, and neither can maintain heterozygosity indefinitely under extreme inbreeding and drift. To fully test the mutational theory of ID, one needs to check whether ID persists in such conditions—approaching a complete absence of genetic variation.

One plausible mechanism to maintain ID in conditions where deleterious mutations have been fixed or eliminated is epigenetic inheritance, broadly defined as the transmission of phenotypic variation via mechanisms independent of DNA sequence changes (Heard & Martienssen, 2014; Jablonka, 2017). For epigenetic mechanisms to create ID, three conditions must be met: (i) heritable epigenetic variation must exist among individuals; (ii) this variation must influence fitness-related traits; and (iii) relatedness between parents or gametes must increase the expression of deleterious epigenetic variants. The latter condition could, for example, emerge from an increase in the occurrence of deleterious homo-epiallelic states—cases where both alleles carry the same deleterious epigenetic mark, an epigenetic equivalent of homozygosity. There are many examples of diseases caused by aberrant epigenetic marks in humans, particularly deleterious epimutations that inappropriately turn off gene expression (Hitchins, 2015; Horsthemke, 2024, 2024). As with classical loss-of-function mutations, their effects are expected to be more severe when both alleles are affected—for instance, when an epimutation inactivates the normally active, non-imprinted allele of a parent-of-origin–imprinted gene (Horsthemke, 2024). For this reason, several models treat epimutations as functionally similar to deleterious recessive mutations, differing mainly in their high forward and backward mutation rates (Geoghegan & Spencer, 2012; Stenøien & Pedersen, 2005). These models usually assume that epigenetic modifications are transmitted independently of genetic variation (“pure” or autonomous epigenetic variants, *sensu*Richards, 2006), in the absence of DNA sequence change (Johannes et al., 2009). Most epigenetic defects associated with human diseases are either caused by cis- or trans-acting mutations, or represent “primary” epimutations arising in somatic tissues that are not transmitted through the germ line (Horsthemke, 2018). However, examples of transgenerational epigenetic inheritance have been documented in invertebrates (e.g., flies, worms) and plants (Heard & Martienssen, 2014; Miska & Ferguson-Smith, 2016). This suggests that epigenetic reprogramming and mark erasure during gametogenesis and zygote development are incomplete in these taxa (Preite et al., 2018), allowing some primary deleterious epimutations to persist across generations and contribute to the standing ID (reviewed by Cheptou, 2024). Moreover, because epimutations arise and revert at much higher rates than genetic mutations (Van Der Graaf et al., 2015), they have the potential to regenerate ID in genetically uniform lines far more rapidly than spontaneous genetic mutation.

Several lines of evidence indeed support a role for epigenetics in ID (Biémont, 2010; Bossdorf et al., 2008; Cheptou & Donohue, 2013; Vergeer et al., 2012). For instance, treatment with a demethylating agent can markedly reduce ID (Vergeer et al., 2012). In maize, performance continues to decline between generations 7 and 11 of selfing lines despite minimal residual heterozygosity, and this deterioration is associated with a genome-wide increase in DNA methylation (Han et al., 2021). This finding indicates that epigenetic change can drive phenotypic deterioration even when further increases in genetic homozygosity are negligible. Likewise, in *Arabidopsis*, heterosis (the reciprocal of ID) is observed when crossing isogenic epi-RILs (i.e., selfing lines differing only in methylation patterns; Dapp et al., 2015). Together, these studies show that epigenetic variation can generate ID-like effects in systems where genetic homozygosity does not vary.

Phenotypic consequences of autonomous epigenetic inheritance have been documented in systems lacking genetic variation (e.g., Bossdorf et al., 2008). In a similar way, if such epigenetic changes play a role in ID, fitness differences should persist among progenies of parents with different degrees of relatedness (self-fertilized broods vs. progenies of sib matings vs. progenies of cousin matings) even when parents are genetically identical (i.e., clonemates). Yet, to date, this approach has not been applied directly.

Here, we tested whether ID can be detected even when genomic heterozygosity is constant. To this end, we generated “outbred clones” by intercrossing two highly inbred lines of the hermaphroditic freshwater snail *Physa acuta*, a naturally outcrossing species in which ID has been repeatedly documented in wild populations. Each line underwent over 28 generations of enforced selfing, effectively eliminating heterozygosity and ensuring near-complete homozygosity. Individuals within each line are thus genetically identical, except for neo-mutations that may have arisen in the last few generations. Their genomes are also likely to have been purged of a substantial fraction of the initial mutation load, as these lines represent survivors from a larger set of inbred lines, many of which went extinct during the inbreeding process. We selected *P. acuta* for this experiment because it can be easily maintained in the laboratory, can be forced to self-fertilize (the most efficient way to eliminate heterozygosity), and is well suited for generating inbred lines.

Crossing individuals from the two inbred lines produced a cohort of heterozygous but genetically identical offspring—our “outbred clone.” From this clone, we set up three types of matings: self-fertilization, sib matings, and cousin matings. These crosses all yielded second-generation offspring with the same expected genomic heterozygosity (ignoring recent mutations), but with different pedigree inbreeding coefficients, reflecting the number of generations since the most recent common ancestor of the gametes. We chose to start with outbred parents (though genetically belonging to the same “outbred clone”) rather than directly with inbred ones. This is because repeated inbreeding and genetic bottlenecks reduce performance through the fixation of deleterious mutations, making highly inbred individuals phenotypically unrepresentative of natural populations (Pujol et al., 2009). Indeed, our inbred lines appear weak compared to their ancestors from natural populations. By contrast, our heterozygous, outbred parents resemble individuals found in nature.

To evaluate whether parental relatedness (selfing, sib mating, or cousin mating) affected offspring performance, we measured four fitness-related traits: juvenile survival, adult body size, probability of laying eggs at the age of 50 days (in isolation), and female fecundity after insemination. Under classical genetic theory, all three types of crosses should yield offspring with equivalent fitness, since they share the same level of genomic heterozygosity. By contrast, a decrease in fitness with parental relatedness (i.e., the typical ID pattern: selfing < sib mating < cousin mating) would imply that ID can be regenerated through heritable variants (mutations or epimutations) that appear in a few generations within the lines so that gametes produced by two sibs or two cousins may carry different variants. We therefore compared our empirical measurements of fitness-related traits with individual-based simulations to evaluate whether plausible genetic mutation rates could account for the observed pattern, or whether substantially faster rates of change—such as those characteristic of epimutations—would be needed.

## Methods

### Study organism and experimental conditions

*Physa acuta* is a widespread simultaneously hermaphroditic freshwater snail that reproduces mostly through cross-fertilization. Snails that have no access to mates can resort to self-fertilization (Henry et al., 2005; Jarne et al., 2010). When a potential mate is present, however, individuals nearly always cross-fertilize (Noël et al., 2016; Pélissié et al., 2012). These snails mate frequently, with unilateral copulations, i.e., they adopt either the male or the female sex role during a given copulation (Wethington & Dillon, 1996), and can store sperm for long periods. Sexual maturity is reached in around 6 weeks under laboratory conditions at 25 °C.

Throughout the experiments––and during the establishment of experimental lines––snails were maintained under standard laboratory conditions (25 °C, a 12:12 photoperiod, water renewed twice a week, and *ad libitum* food in the form of boiled ground lettuce). Each individual was kept in a 75-ml plastic box. In addition, albino lines of *P. acuta* are maintained in our laboratory. Albinism (a single-locus recessive trait) can be used to assess paternity success of wild-type focal individuals because wild-type offspring produced by albino mothers must have a wild-type father.

### Field sampling and breeding design

The first step was to collect snails from the field and generate two inbred lines through continuous self-fertilization over many generations. Self-fertilization was enforced by keeping mature individuals isolated until they lay eggs, usually 2 or 3 weeks after the age at which they normally start laying eggs when they have mates available (Tsitrone et al., 2003). The two ancestral wild-caught snails were part of a larger sample taken from a wild population at the “Pont Romain” in the Lirou River (43°43′47.0″N, 3°49′50.4″E), located close to the village of Les Matelles, 15 km north of Montpellier (France) on October 23, 2013. The initiation of 44 inbred lines is described in Janicke et al. (2022), who used them for an experiment after the third generation of self-fertilization. A subset of the lines were propagated by continuous self-fertilization until May 2019, a time at which they had all reached at least 25 generations of selfing. The inbreeding coefficient at these stage is therefore expected to be, at least, 1–1/2^25^ = 0.99999997. To evaluate whether inbred individuals were still capable of cross-fertilization, we performed a preliminary analysis described in the Supplementary Material. Once confirmed that individuals from inbred lines were fully capable of performing cross-fertilization, we conducted the main assay.

### Estimating inbreeding depression in outbred clones

**Figure 1.**
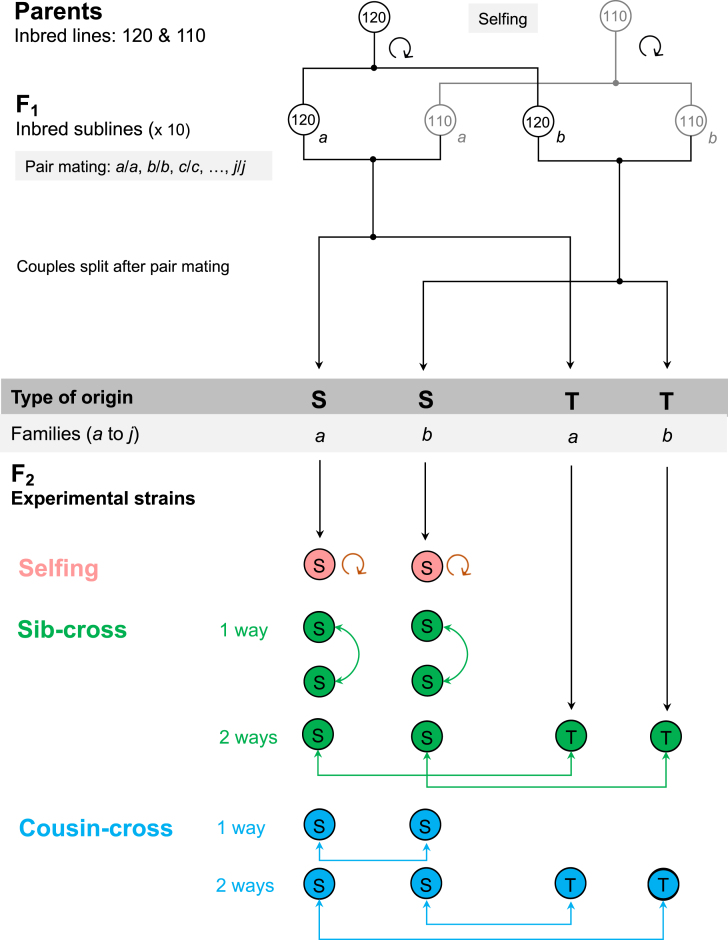
Pedigree of the F_2_ snails used in this study, and how they were crossed to produce the F_3_ on which fitness traits were measured. Parents were two individual snails extracted from independent inbred lines (named 120 and 110) derived from wild-caught ancestors through 28 or 29 successive generations of self-fertilization in the laboratory, respectively. F_1_ individuals (inbred sublines) resulted from self-fertilization of parents, and were also homozygous. The F_1_ pair crossing yielded a set of individuals that constituted the experimental strains (F_2_ snails), which are all heterozygous and genetically identical to one another, but differ in their parental type of origin (S and T). F_2_ snails from the S type had their mothers from the 120 line and their fathers from the 110 line, and *vice versa* for those from type T. The experimental strains were constituted by 10 families (*a* to *j*) per type of origin.

We selected two inbred lines named 120 and 110. At this stage, these lines had undergone 28 and 29 generations of self-fertilization, respectively. The residual heterozygosity is expected to be <4.10^−9^ times that of the wild-caught individuals, ignoring recent mutations. As a first approximation, we therefore consider inbred lines as fully homozygous clones. The pedigree and the mating protocol described below are depicted in Figure 1 and Figures S1–S6.

#### First step: obtaining F_2_ snails

One individual from each inbred line, 120 and 110, was isolated and forced to self-fertilize to produce the F_1_. F_1_ offspring were isolated at day 28 after egg-laying (still immature) and then raised in isolation until maturity for three more weeks. We assigned a code letter to the F_1_ siblings for identification (120_a_, 120_b_, 120_c_, and so on; and, 110_a_, 110_b_, 110_c_, and so on), constituted inter-line pairs of F_1_ individuals (e.g., 120_a_ × 110_a_, 120_b_ × 110_b_), and let them mate for 1 week. To differentiate the 120 snail from its 110 partner in each pair, we added small dots of harmless car-body paint of different colors on their shell (Henry & Jarne, 2007). After mating, F_1_ individuals were re-isolated to lay F_2_ eggs. We successfully collected and raised the resulting F_2_ hatchlings for 10 families *a* to *j* (Figure 1).

F_2_ snails belonged to two different types, hereafter type S and type T. The S-type F_2_ offspring were laid by a mother from the 120 line, and sired by fathers from the 110 line, while T-type offspring came from the reciprocal combination (mother 110–father 120). As these two types are present within all 10 families *a* to *j*, we end up with 20 categories (S*_a– j_* and T*_a_*_–_*_j_*), each represented by several offspring. Note that irrespective of the category they belonged to, all F_2_ were expected to be heterozygous and genetically identical to one another (i.e., they form a heterozygous clone; Figure 1; Figure S1). We proceeded until we obtained at least 40 F_2_ descendants of type S and 10 of type T for each family. This procedure sometimes required taking several successive clutches or even re-mating the same pair to stimulate egg-laying. These descendants were raised and isolated before maturity, as previously mentioned, and kept until fully mature and ready for mating. Then, we used them to generate different types of F_3_ snails.

#### Second step: self-fertilization, sib, and cousin pair-crosses

We submitted each F_2_ snail to one of three mating treatments: (i) selfing, (ii) sib-cross, and (iii) cousin-cross. Sib- and cousin-crosses were themselves divided into two subtypes depending on direction of exchange of genetic material (one and two ways), giving five treatments in total. We explain this in more detail in the following.

**Figure 2.**
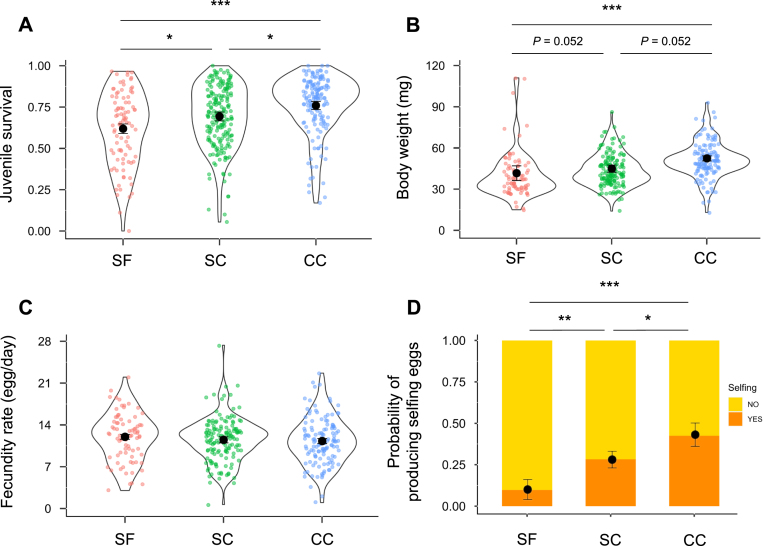
Fitness traits measured on F_3_ individuals of the hermaphrodite snail *Physa acuta*. (A) Juvenile survival (proportion of eggs that become juveniles alive after 14 days). (B) Body weight at 50–53 days (the mean of the two measures, before and after pairing the focal snail with a virgin albino partner). (C) Female fecundity rate after insemination (number of eggs per day produced by the focal snail). (D) Probability of having produced self-fertilized eggs at 50 days just before pair mating. Mating treatment effect sizes (difference between two types divided by its *SD*) were tested using post hoc tests with the Holm–Bonferroni correction for multiple testing (^∗^*p* < 0.05; ^∗∗^*p* < 0.01; ^∗∗∗^*p* < 0.001). SF, selfing; SC, sib-cross; CC, cousin-cross. Black dots are mean values ± 1 *SE*. We did not separate one-way and two-way crosses in the SC and CC category as differences between them were small and not significant (see Tables S1–S3).

The first treatment included S-type individuals that were forced to self-fertilize (selfing, Figures 1; Figure S2). Thus, 10 F_2_ S-type individuals per family (i.e., S*_a_*_,1–10_, S*_b_*_,1–10_, . . ., S*_j_*_,1–10_) were isolated and eggs were collected 3 days after we detected the first clutch. The second treatment consisted in pair-crossings between two S-type snails from the same family (called “one-way sib-cross”). So, for instance, in family *a*, we crossed individuals S_*a*,11_ . . . S_*a*,20_, according to the following pairs: S*_a_*_,11/_S*_a_*_,16_, S*_a_*_,12/_S*_a_*_,17_, and so on (and similarly for S*_b_* to S*_j_*) (Figure 2; Figure S3). Note that in these crosses the two parents are full sibs and they have the same mother and the same father. The third treatment consisted in pair-crossings within the same family (e.g., family *a*) but between S and T individuals, which we defined as “two-way sib cross.” For instance, we paired snails from S*_a_* (numbered from 21 to 25) with snails from T*_a_* (numbered from 1 to 5), which gave us the following combinations: S*_a_*_,21/_T*_a_*_,1_, S*_a_*_,22/_T*_a_*_,2_, and S*_a_*_,23/_T*_a_*_,3_, and so on. The two parents in these crosses are full sibs but not obtained in the same way (the father of one is the mother of the other and reciprocally; Figure 2; Figure S4). The fourth treatment involved pair-crossings between two F_2_ S-type snails (numbered from 26 to 35) from different families, which we called the one-way cousin cross (the two parents are cousins, belonging to the same type). Because of insufficient individuals in families *h* and *j*, we were able to do this for four pairs of families. The resulting combinations were S*_a_*_,26–35/_S*_b_*_,26–35_, S*_c_*_,26–35/_S*_d_*_,26–35_, S*_e_*_,26–35/_S*_f_*_,26–35_ and S*_g_*_,26–35/_S*_i_*_,26–35_ (Figure 2; Figure S5). Finally, the fifth treatment consisted in pair-crossings between snails of the S-type (36 to 40) and of the T-type (6 to 10) from different families, which we defined as the two-way cousin cross. The combinations were S*_a_*_,36–40/_T*_b_*_,6–10_, S*_c_*_,36–40/_T*_e_*_,6–10_, or S_d,36–40/_T*_f_*_,6–10_, and so on (Figure 2; Figure S6). When necessary, we added different color dots of car-body paint on each individual’s shell to differentiate snails from each family and origin.

After pairing for 3 days, mated snails were re-isolated to lay eggs for 3 days. We collected two successive F_3_ clutches for each F_2_ parent, by transferring the parent to a new box after 3 days. These F_3_ clutches constitute the generation on which we evaluated fitness traits.

#### Third step: assessing and comparing fitness components in F_3_ snails

Eggs were counted in all F_3_ clutches right after collection and live juveniles were counted in the boxes 14 days later. We estimated juvenile survival as the ratio of juveniles over eggs. Next, we randomly selected one of the surviving F_3_ individuals per box and raised it in isolation until age 50 days. At this age we weighed each snail and determined whether it had initiated egg laying or not. Given that each individual was kept in isolation, this egg-laying represents self-fertilization. Then, we paired it with a same-age albino partner for 3 days. Once inseminated, we isolated the F_3_ individual once more, weighed it a second time, and allowed it to lay eggs for 3 days. Finally, we counted these eggs that represent female fecundity of F_3_ under cross-fertilization conditions. We therefore have four traits on the F_3_: juvenile survival, body mass at 50–53 days (the mean of the two measures), self-fertilization ability at 50 days (yes/no), and female fecundity after insemination at 54–57 days (number of eggs).

### Heterozygosity

We genotyped a subset of the F_2_ and F_3_ individuals of different categories using seven microsatellite loci chosen because preliminary data indicated that the inbred lines 110 and 120 were fixed for different alleles. The loci were AF108762, AF108764 (Monsutti & Perrin, 1999), Pac1, Pac2 (Sourrouille et al., 2003), Pasu1-2, Pasu1-9, and Pasu1-11 (*cf*. Escobar et al., 2008) grouped into two multiplexes. We expected that the F_2_ would be fully heterozygous (except for a portion of misreadings), whereas in the F_3_ generation, half of the loci would, on average, exhibit heterozygosity. We assessed the number of heterozygous and homozygous loci for each individual. However, it was inevitable that a small fraction of the genotypes remained unamplified, leading to missing data. Moreover, in microsatellite readings heterozygous genotypes are frequently misconstrued as homozygous, i.e., one allele behaves as partly “dominant” (David et al., 2007). To avoid any bias, we kept the readings blind to the sample’s origin. Also, we refrained from making corrections, even in instances where the reading error was evident (e.g., one homozygous locus and all other loci heterozygous in an F_2_).

### Statistical analyses

All analyses were performed with *R* 4.0.2 packages *lme4* (Bates et al., 2015) and *nlme* (Pinheiro et al., 2017) by fitting generalized linear mixed models for binomial variables (juvenile survival, ability to self-fertilize, proportion of heterozygous loci) or linear mixed models for Gaussian continuous variables (body weight, female fecundity). Our linear models included as fixed effects: (i) mating treatment (selfing, sib-cross, and cousin-cross), (ii) the cross type (one vs. two ways) when appropriate (i.e., for sib- and cousin-cross), and (iii) the maternal type (S vs. T) within two-way crosses (recall that in the selfing and one-way crosses all mothers are S). We proceeded by first running separate analyses within the sib-mating and cousin-mating treatments in order to test the mating treatment and maternal type effects. As these effects were not significant, we pooled all subcategories within the sib-cross and cousin-cross treatments in order to test the mating treatment effect using the whole dataset. In each model we added an appropriate structure of random effects to account for various levels of replication within each level of fixed factors (e.g., identity of the maternal family: *a, b, c*; pair of parental families: *a*/*b, c*/*d*; identity of the mother: 1 to 35; and/or of the parental pair: 21/1, 22/2 described for each model in the results). Observation number was also added as a random factor in the binomial models on juvenile survival in order to account for overdispersion (Browne et al., 2005; Elston et al., 2001). Tests of fixed effects were made using LRT (likelihood-ratio tests, which compare the likelihood of models with and without the factor of interest) keeping all random effects present in the model. Random effects were tested using LRT with the corrections indicated by Zuur et al. (2009, chap. 5, pp. 123–125). Post hoc tests were performed when the treatment effect was significant using Holm–Bonferroni correction for multiple testing to test effect sizes among different treatments.

### Simulations

We made individual-based simulations to evaluate whether the observed changes in fitness between the three type of mating treatments (selfing, sib-cross, and cousin-cross) could be compatible with a purely genetic model of ID (i.e., deleterious-mutation based). Each individual genome was modeled as a single chromosome on which deleterious mutations were located. The deleterious mutations were of two types: small effect and large effect (quasi-lethal), each type with its own rate, degree of recessivity, and effect. We modeled both the process of extraction from a large population and the propagation of two inbred lines over 28 generations with the following events at each generation: mutation, meiosis, recombination, selection, and drift within each line. Then, we simulated the creation of F_1_, F_2_, and the three types of crosses giving the F_3_ as in our experiment. The output of each simulation was a set of three average fitness values for each of the three types of F_3_. For each set of parameters, 1,000 independent replicates were made to produce expected distributions of the relative fitnesses of the three categories. We conducted several series of simulations. In a first set, mutation rates were constrained to match the empirical ID observed in natural populations (ID ≈ 0.5 for juvenile survival). Within this constrain, we explored a wide range of parameter values, including the relative contribution of large- versus small-effect mutations, their average fitness effects and dominance coefficients, and the degree of linkage among loci. In a second set, we increased the mutation rate beyond values compatible with natural levels of ID. Finally, we implemented simulations designed to mimic the expected behavior of epimutations rather than mutations, by assuming a very high rate of spontaneous appearance of new variants (up to 100 times reference mutation rates) combined with high reversion rates (5%–50%), representing the “resetting” of epigenetic states during gametogenesis or embryogenesis (Stenøien & Pedersen, 2005). These latter simulations were constrained to match either the full or half of the natural values of ID, reflecting the possibility that epimutations contribute only partially to the ID. The details on the simulations are explained in *Simulation Details* in the Supplementary Material.

## Results

Table 1 and Figure 2 summarize our main results, while linear models are reported in detail in Tables S1–S3.

**Table 1.** Summary of means (± *SE*) and statistical significance of the mating treatment type effects on fitness traits measured on F_3_ individuals of the hermaphrodite snail *Physa acuta*.

Variable	Mating treatment			Treatment effect	Treatment comparison	Effect size
	Selfing (SF)	Sib-cross (SC)	Cousin-cross (CC)			
Juvenile survival	0.62 ± 0.03 (93)	0.69 ± 0.03 (193)	0.76 ± 0.02 (153)	χ^2^_2_ = 15.40; *p* **< 0.001**	SF vs. CC	−4.04***
					SF vs. SC	−2.74*
					SC vs. CC	−2.33*
Body weight	41.6 ± 5.3 (82)	44.9 ± 2.6 (152)	52.3 ± 1.8 (134)	χ^2^_2_ = 10.06; *p* **= 0.007**	SF vs. CC	−2.98**
					SF vs. SC	−1.94
					SC vs. CC	−2.23
Fecundity rate	12.0 ± 0.4 (79)	11.5 ± 0.5 (146)	11.3 ± 0.4 (127)	χ^2^_2_ = 1.53; *p* = 0.466	—	—
					—	—
					—	—
Probability of producing selfed eggs at the age of 50 days	0.10 ± 0.06 (82)	0.28 ± 0.05 (152)	0.43 ± 0.07 (134)	χ^2^_2_ = 19.69; *p* **< 0.001**	SF vs. CC	−4.25***
					SF vs. SC	−3.09**
					SC vs. CC	−2.0 *

*Note*. Number of observations is indicated in parentheses. In this table, as in Figure 2, we did not distinguish, within each mating treatment, the different possible types of crosses (one-way vs. two-way, see Figure 1) because they were not statistically different. Detailed results are reported in Tables S1–S3. Treatment means (± *SE*) are reported on the observed (natural) data scale for the four traits (proportion of eggs surviving, body weight in milligrams, eggs laid per day, and proportion of laying individuals, respectively). Statistical analyses were conducted using generalized linear mixed models on the appropriate model scale (Binomial errors with logit link for juvenile survival and probability of producing selfed eggs; Gaussian errors with identity link for body weight and fecundity), including random effects to account for the experimental design (not shown here; see the *Methods* section and detailed results in Supplementary Materials and Tables S1–S3). Mating treatment effect sizes (estimated mean difference on the model scale divided by its SD) were tested using post hoc comparisons with the Holm–Bonferroni correction for multiple testing. *p < 0.05; ^∗∗^*p* < 0.01; ^∗∗∗^*p* < 0.001 after correction.

Juvenile survival significantly differed across treatments. The highest survival was in the offspring of cousin-cross (average 0.76) whereas survival in the selfing and sib-cross treatments was reduced by 18% and 9%, respectively, compared to cousin-cross (Table 1 and Figure 2A). Body weight showed a similar pattern, the cousin-cross treatment had the highest mean, with a reduction of 20% and 14% in the self-fertilization and sib-cross treatment, respectively (Table 1 and Figure 2B). Despite this difference in body weight, there was no significant effect of parental relatedness on female fecundity at 53 days, after insemination (Table 1 and Figure 2C). In contrast, we found a strong treatment effect on the probability of having produced eggs by self-fertilization at the age of 50 days. This probability was the highest in cousin-cross treatment and it decreased by 77% and 35% in the selfing and sib-cross treatments, respectively (Table 1 and Figure 2D).

**Figure 3.**
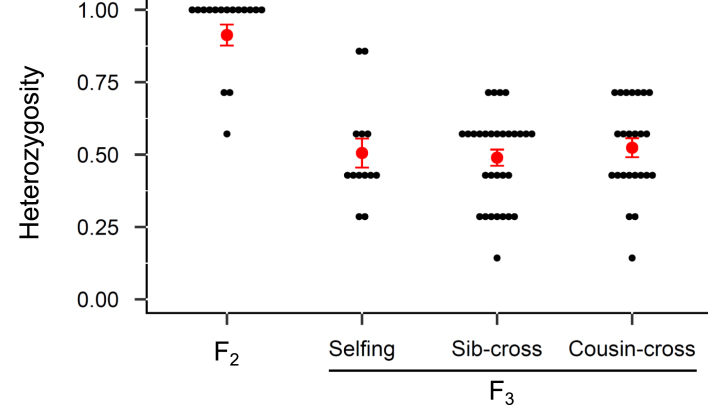
Distributions of multilocus heterozygosity for a subset of F_2_ and F_3_ individuals of different categories using seven microsatellite loci (AF108762, AF108764, Pac1, Pac2, Pasu1-2, Pasu1-9, and Pasu1-11) chosen because preliminary data indicated that the inbred lines 110 and 120 were fixed for different alleles. The microsatellites were grouped into two multiplexes. Overall, F_2_ individuals were fully heterozygous (with a few exceptions) whereas in the F_3_ heterozygosity was spread around 0.5 within each mating treatment. Mean values (± 1 *SE*) are shown with points and error bars.

We genotyped F_2_ individuals, which represent inter-line crosses in our design (their offspring are F_3_), using microsatellites fixed for alternative alleles in the two inbred lines. As expected, almost all F_2_ were fully heterozygous, with rare exceptions where one or two loci appeared homozygous in a few individuals, likely due to genotyping errors (Figure 3). In the F_3_ generation, we found no significant differences in heterozygosity among mating treatments (*χ*^2^_2_ = 0.475, *p* = 0.789, binomial model on the proportion of heterozygous loci). The multilocus heterozygosity distribution within each treatment was centered around 0.5, consistent with expectations for F_2_ × F_2_ progeny: selfing (*H_O_* = 0.51 ± 0.05), sib-cross (*H_O_* = 0.49 ± 0.16), and cousin-cross (*H_O_* = 0.52 ± 0.16; Figure 3).

**Figure 4.**
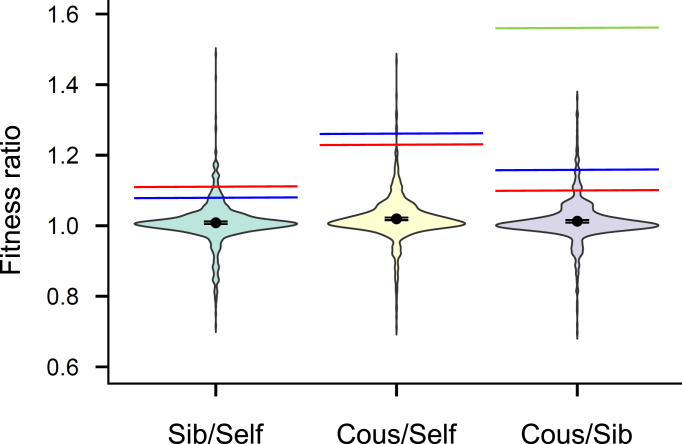
Violin plots of the distribution of fitness ratios from 1,000 simulations of our experiment, using the reference parameter set, compatible with available data on ID in *Physa acuta* (see the *Methods* section and *Simulations* in the Supplementary Material). Parameters include the length of the genome (*L* = 10 Morgans), the dominance coefficient and effect size of semi-lethals (*h*_1_ = 0.02, *s*_1_ = 0.9), and those of small-effect mutations (*h*_2_ = 0.2, *s*_2_ = 0.05), with both mutation types contributing to a total inbreeding depression of *d =* 0.5 in natural populations (*d*_1_ = 0.3 for semi-lethals and *d*_2_ = 0.2 for small-effect mutations). Black dots represent the mean fitness ratio, with 95% confidence intervals (CI) shown. The observed ratios for juvenile survival (red line), body mass (blue line), and the proportion of fertile individuals at 50 days (green line) were for sib/self: 1.11, 1.08, and 2.80 (not shown, out of range); for cous/self: 1.23, 1.26, and 4.30 (not shown); and for cous/sib: 1.10, 1.16, and 1.54, respectively. Simulations with different parameter sets are in Figure S7.

We conducted simulations replicating our experimental design to determine whether the observed differences between treatments could result from incomplete fixation, linkage, or spontaneous mutation. The mutational parameters were selected to align with previous estimates of ID in natural, outbred populations of *P. acuta* (Escobar et al., 2008). The simulations showed that offspring fitness from full sib- and cousin-crosses was only slightly higher than that from the selfing treatment, with differences of 0.8% and 2.0%, respectively—well below the observed differences in juvenile survival, body weight, and the probability to lay eggs in isolation (Figure 4). We tested various parameter sets compatible with naturally observed levels of ID, adjusting linkage, contributions of lethal mutations, and selection or dominance coefficients, but these had minimal effects on the results. The largest predicted difference in fitness between cousin-cross, sib-cross, and self-fertilization occurred when the source of ID was driven quasi-exclusively by mutations to semi-lethals (*d*_1_ = 0.49, *d*_2_ = 0.01) and when these mutations were nearly fully lethal (*s*_1_ = 0.99). However, even under these extreme conditions, average fitness differences never exceeded 5% (see Figure S7). While individual replicate runs varied considerably within each parameter set, the mean predictions consistently fell well below the observed ratios in all situations. Simulations could match the experimental results only in two situations. First, when the mutation rate was artificially increased by approximately 10- to 12-fold beyond the values compatible with the levels of ID estimated for natural populations of *P. acuta* (Figure S8). Second, when we used parameters typical of epimutations rather than mutations, i.e., when we introduced high reversion rates (of the order 0.1) that compensate very high forward (epi)mutation rates, so as to remain compatible with ID estimated in natural populations (Figure S9).

## Discussion

Our study reveals substantial ID in the progeny of experimental lines: juvenile survival, body mass, and self-fertility declined with increasing parental relatedness (selfing < sib-crosses < cousin-crosses), while female fecundity after mating showed no significant differences. The amount of ID in juvenile survival (0.18) and body mass (0.20) in the selfing treatment, relative to the cousin cross, is lower than typical values reported for outbred and self-fertilized offspring of wild-caught *P. acuta*, where ID estimates are approximately 0.55 for juvenile survival (Escobar et al., 2008; Janicke et al., 2013) and 0.38 for body mass (Janicke et al., 2013). Yet, these values still represent a non-trivial fraction (∼1/3–1/2) of natural estimates for these traits. This pattern conflicts with the expectation that extreme inbreeding should produce F_3_ categories with essentially identical genome-wide heterozygosity; only spontaneous mutations arising in the last few generations could make them differ. However, individual-based simulations accounting for these mutations fail to reproduce the magnitude of differences we observed, unless mutation rates are inflated to implausibly high values (>10 × the value compatible with naturally occurring levels of ID; see the *Results* section, Figure 4; Figures S7 and S8). Together, these empirical and simulation results suggest that non-genetic mechanisms—most notably epigenetic inheritance—can maintain persistent ID in genetically uniform lines. Below, we discuss potential biases and alternative interpretations in more detail.

A first potential concern is that some F_2_ or F_3_ individuals might have failed to cross-fertilize after 28 generations of enforced selfing, which could artificially generate differences among treatments. However, two lines of evidence dispel this possibility. First, additional crosses confirmed that individuals from both inbred lines could act as both male and female partners (see Supplementary Material: *Outcrossing ability of inbred lines*). Second, microsatellite genotyping supported the expected patterns: F_2_ offspring were fully heterozygous at diagnostic loci, and the three classes of F_3_ offspring all exhibited an average multilocus heterozygosity of 0.5. The occasional apparent homozygosity at isolated loci is best explained by genotyping errors, not by undetected selfing. For comparison, in classical ID experiments, selfed offspring typically exhibit about half the heterozygosity of outcrossed ones—a pattern clearly absent here.

In the absence of variation in heterozygosity, maternal effects could in principle generate differences between self-fertilized and cross-fertilized broods. However, because mothers were randomly assigned to all treatments, variation in maternal quality cannot explain the results. Moreover, maternal effects on offspring fitness are known to be weak in *P. acuta*. For example, outcrossed offspring of inbred mothers do not perform worse than those of outbred mothers (*cf*. Janicke et al., 2013; Noël et al., 2016), even though the inbred mothers themselves are less fit than outbred ones. Another possibility is that mothers may distinguish self-fertilized from outcrossed eggs and bias their resource allocation accordingly (Cheptou, 2024; McCarthy & Sih, 2008). Yet, this mechanism cannot account for the fitness differences we observed between sib- and cousin-crosses. Finally, we must consider the possibility that interactions between the nuclear genome and maternally transmitted cytoplasmic genes (e.g., mitochondrial genes) might generate lineage-specific variation in fitness—i.e., differences between the T- and S-type offspring in our design. However, T and S types exhibited very similar performance under outcrossed conditions (Tables S2 and S3), and the ID pattern persisted when we analyzed only the S-type progeny, which share a common maternal background. Taken together, these lines of evidence indicate that neither maternal effects nor cytonuclear interactions are likely to explain the observed ID.

Our simulations indicated that 28 generations of selfing in the history of our inbred lines were sufficient to eliminate ancestral heterozygosity for deleterious mutations—either by fixation or loss—as suggested by our microsatellite data. However, seven microsatellite loci capture only a small fraction of the genome, and two mechanisms could, in principle, maintain residual heterozygous load elsewhere in the genome: (i) physical linkage (Lande et al., 1994; Waller, 2021) and (ii) spontaneous mutations. Regarding the first point, heterozygous pseudo-overdominant blocks (Waller, 2021) might persist in inbred lines if deleterious alleles happen to be tightly linked. However, even when we imposed an unrealistically small genome size of 1 Morgan (while *P. acuta* has 19 pairs of chromosomes), our simulations failed to reproduce the magnitude of ID observed (see Figure 4; Figure S7). This reflects the fact that our experimental design involved far more extreme and prolonged inbreeding than most empirical studies where persistent heterozygosity or ID has been reported (Chelo et al., 2019; Latter, 1998; Rumball et al., 1994; Williams et al., 2016). Under such strong inbreeding, residual heterozygosity could persist only in chromosomal fragments harboring deleterious mutations acting as balanced lethals (Wang & Hill, 1999). In that scenario, heterozygosity—and thus fitness—would be expected to differ between selfed and outcrossed F_3_ offspring, as F_2_ individuals could inherit distinct chromosomal fragments from their F_1_ parents. By contrast, no fitness difference should arise between sib- and cousin-crossed F_3_ individuals, because all F_1_ parents would carry the same heterozygous balanced-lethal segment. This prediction is inconsistent with our results, making linkage among deleterious mutations an unlikely explanation for the fitness variation observed in our highly inbred lines.

With regard to spontaneous mutation, our simulations indicate that the mutations most likely to generate detectable fitness effects under our design are strong-effect mutations (i.e., semi-lethals). When segregating in a heterozygous state in highly selfing lines, such mutations could confer a fitness advantage to sib- or cousin-crossed offspring relative to selfed offspring. However, their persistence is very short: homozygous lethals are unviable, so each generation removes one-third of lethal alleles, yielding a half-life of only two to three generations. Mutations of milder effect persist for an even shorter time as heterozygotes, because homozygous carriers are not fully unviable and such alleles can therefore become fixed rather than eliminated. Consequently, any standing heterozygous mutational load in selfing lines must originate from only a few recent generations. However, even under the most favorable scenario––namely an unusually high proportion of lethal mutations––this mechanism remains unlikely to explain our results. In our simulations, we assumed an average per-haploid genome rate of spontaneous semi-lethal mutations of *U*_1_ = 0.028. Although direct estimates of *U*_1_ are unavailable for *P. acuta*, this value is close to empirical estimates for *Drosophila melanogaster* (0.003–0.017 for the large chromosomes 2 and 3, and 0.006–0.034 for the full genome; Charlesworth et al., 2004; Fry et al., 1999; Sharp & Agrawal, 2012). Across this empirically plausible range of mutation rates (*U*_1_ ≈ 0.003–0.034 per genome per generation), simulated sib- and cousin-crosses showed, on average, at most a slight viability advantage (<5%) over selfed offspring. This predicted effect is consistent with the short persistence time of recent semi-lethal mutations (two to three generations) but remains far smaller than the differences observed in juvenile survival. As expected for processes driven by very recent mutations, fitness ratios showed substantial stochastic variation across simulations (Figure 4), particularly when lethal mutations were frequent. This variability is expected, given the stochastic nature of recent mutations in the lines just a few generations before the experiment. Although the observed survival estimates remain improbable under a purely mutational model, a small fraction of simulations occasionally yield survival ratios comparable to those observed (3.8% for sibling/selfing, 0.8% for cousin/selfing, and 6% for cousin/sibling; Figure 4). Definitively excluding a purely mutational explanation for these differences would require replication using independent line pairs of inbred lines.

Body size and the proportion of self-fertile individuals at the age of 50 days also showed strong ID, with ratios even higher than those observed for juvenile survival. These later-expressed traits typically contribute less to overall ID than juvenile survival in wild-caught *P. acuta*. Moreover, they are less susceptible to purging (Escobar et al., 2008; Janicke et al., 2013; Noël et al., 2016), suggesting that they are influenced predominantly by numerous small-effect mutations rather than by semi-lethal ones. For these traits, simulation parameters should therefore shift toward lower per-generation genomic mutation rates and a higher proportion of small-effect mutations. Yet, such parameter changes further reduce residual heterozygosity within the lines, thereby pushing simulated ID even farther from the values observed experimentally. The consistency of this mismatch across multiple traits reinforces the conclusion that the magnitude and persistence of ID detected in our genetically uniform lines cannot be explained by classical mutational models. Indeed, reproducing the observed patterns would require an unrealistically large increase in mutation rates—exceeding a 10-fold elevation—in our inbred lines relative to the original populations (see Figure S8). While individuals in poor physiological condition, as expected under severe inbreeding, can exhibit modestly elevated mutation rates (Agrawal & Wang, 2008; Sharp & Agrawal, 2012), the magnitude of increase required to explain our results remains implausible. In *Drosophila*, for instance, individuals carrying multiple deleterious alleles accumulate spontaneous mutations at rates no more than twice those of healthy individuals, and this increase does not extend to lethal mutations (Sharp & Agrawal, 2012).

One possible explanation for the mismatch between our experiment and the simulations is that the experiment was conducted in a laboratory environment. The expression of ID and the effects of deleterious mutations are known to be affected by environment, sometimes in unpredictable ways (Armbruster & Reed, 2005). In our simulations, mutation rates were constrained so that mutation-selection equilibrium produced ID = 0.5, matching the value observed in G_0_ or G_1_ individuals from natural populations of *P. acuta* raised in a laboratory environment. If the same mutational load produced, for example, ID = 0.7 in the natural environment, higher mutation rates would be required to generate it. However, the only available study of ID in *P. acuta* in the natural environment found that, for two traits (adult growth and adult survival), ID was higher in the laboratory than in nature (Henry et al., 2003). This pattern may depend on the specific environment and season studied, and additional studies are needed. Nevertheless, ID in nature is unlikely to be high enough to cause a 10-fold (or greater) underestimation of mutation rates, which would be necessary to reconcile the simulations with our experimental observations.

Our results therefore point either to an unprecedented acceleration of mutational processes or, more plausibly, to a fast-renewing source of ID distinct from mutation. One candidate mechanism is the accumulation of deleterious epimutations—heritable changes in gene expression mediated by DNA methylation, small RNAs, or histone modifications. In maize, for instance, inbred lines display a steady decline in fitness coupled with a genome-wide increase in DNA methylation over successive generations of selfing (Han et al., 2021). This pattern has been attributed to incomplete or disrupted resetting of DNA methylation during gametogenesis. However, such a mechanism is unlikely to account for our results, since the founders (F_2_) of our experimental lines were all highly outbred individuals, a condition expected to largely restore baseline methylation states (Han et al., 2021).

Instead, our findings are more consistent with the idea that ID arises from increased epigenetic similarity between uniting gametes. Epigenetic marks can be transmitted across generations with incomplete fidelity, suggesting that epigenetic information is carried by gametes through mechanisms that remain only partially understood, notably those involving small RNAs (e.g., Cecere, 2021). Consequently, gametes produced by the same individual are expected to share more similar epigenetic states than gametes produced by siblings or cousins, generating a gradient of decreasing epigenetic similarity as parental relatedness declines. This gradient would arise because the number of meiotic cycles and epigenetic “resetting” events separating gametes increases from selfing to sib- to cousin-crosses, thereby reducing the probability that both gametes carry the same deleterious epimutation (i.e., a homo-epiallelic state; Figure 1; Figures S2–S6). Consistent with this interpretation, simulations incorporating high reversion rates (representing epigenetic resetting between generations) and very high rates of appearance of deleterious variants (Figure S9) reproduce the patterns observed in our experiment—patterns that are not recovered under purely mutational models. These simulations should nonetheless be regarded as exploratory, given the currently limited empirical knowledge of the fitness effects of epimutations and their modes of inheritance in invertebrates.

Several lines of empirical evidence support the hypothesis of an epigenetic component of the inbreeding load across diverse taxa. In *Arabidopsis*, crosses between isogenic epi-RILs that differ only in methylation patterns exhibit heterosis—the reciprocal of ID (Dapp et al., 2015). In addition, selection for fitness-related traits (plant diameter and height) among these epi-RILs shifts the genomic methylation landscape toward that of the wild type, suggesting that hypomethylated regions can act as deleterious heritable variants (Pujol et al., 2025). In mollusks—including freshwater pulmonates closely related to *Physa*—environmentally induced methylation changes can alter growth, development, and gene expression, and some of these effects persist across generations (Fallet et al., 2020; Luviano et al., 2021). In *Biomphalaria glabrata*, for instance, exposure to DNA methyltransferase inhibitors induces methylation changes that alter growth and gene expression, with effects persisting into the next, untreated generation (Luviano et al., 2021). Beyond their direct phenotypic effects, methylation variants may also influence the mobilization of transposable elements (TE), which are typically silenced by methylation. In this case, even if epigenetic variants tend to revert rapidly to wild type when methylation-control machinery is intact, they could act as transient triggers of more stable genetic variants associated with novel phenotypes (Baduel & Colot, 2021). The boundary between epigenetic and genetic contributions to phenotypic variation is therefore not clear-cut. However, collectively, these studies show that both hyper- and hypomethylation, including TE-linked epigenetic variation, can generate heritable phenotypic effects over one or several generations. This provides a plausible mechanistic basis for the fast-renewed load required to explain the patterns observed in our experiment.

A key distinction between epimutations and genetic mutations is that epimutations arise but also revert at much higher rates. Whereas deleterious genetic mutations are primarily eliminated from large natural populations through selection (purging), epimutations are additionally subject to several forms of unfaithful transmission. These include epigenetic reprogramming during embryogenesis and paramutation—a process akin to gene conversion, whereby the epigenetic state of one chromosome can overwrite that of its homolog—both of which can efficiently reduce epigenetic heterozygosity (Geoghegan & Spencer, 2013; Van Der Graaf et al., 2015). We represented this unfaithful transmission in our models using high reversion rates (5%–50% per generation). Although still highly speculative, these simulations illustrate how a rapid turnover of deleterious epimutations could regenerate inbreeding load within inbred lines over only a few generations, while simultaneously limiting its long-term accumulation in large populations. At present, we have no clear idea of the relative contributions of epigenetic versus genetic components to ID in natural populations. Nevertheless, the existence of an epigenetic component would have several important consequences. First, complete purging of the inbreeding load may be impossible if the epigenetic component is regenerated within a few generations. Second, a fraction of the load may also escape fixation, owing to recurrent reversion toward the epigenetic wild-type state. Such dynamics are expected to be particularly relevant in bottlenecked captive or wild populations, or in populations undergoing shifts in mating system from outcrossing to inbreeding. In these contexts, the inbreeding load may evolve on two distinct timescales: a slow one for deleterious genetic mutations, which can go to fixation or be purged, and a faster one for epigenetic variants, for which fixation and purging are inherently limited.

In summary, our study demonstrates that ID—typically expressed as reduced offspring fitness with increasing parental relatedness—can arise even in genetically uniform populations. The magnitude of ID we observed, affecting both juvenile and adult traits, represents a substantial fraction of that reported in natural, genetically polymorphic populations for the same traits. Although spontaneous mutations continuously arise and may create minor genetic differences among individuals, they appear insufficient to account for the strength, consistency, and concordance of effects observed across three independent traits. While genetic mutations undoubtedly contribute to ID in natural populations, it would be premature to assume they explain its full extent—particularly given the long-recognized difficulty of completely eliminating ID through purge or fixation. Our findings support the view that epigenetic mechanisms may play a more pervasive role in ID than previously appreciated, aligning with growing evidence that epimutations and epigenetic inheritance generate heritable phenotypic variation across taxa. Our support for the epigenetic hypothesis is an inference by elimination—arising from patterns that appear incompatible with purely mutational mechanisms rather than from the direct identification of causal epimutations. Yet, pinpointing all causal variants underlying ID is notoriously difficult, whether genetic or epigenetic. Moreover, any heritable effects that persist in the absence of genetic variation must, by definition, be considered epigenetic, irrespective of their molecular basis. Future work spanning a broader range of genotypes, species, and ecological contexts, combined with investigations of the underlying molecular mechanisms, including potential TE mobilization, will be essential to assess the generality and evolutionary significance of epigenetically mediated ID.

## Supplementary Material

qrag008_Supplemental_File

## Data Availability

The data that support the findings of this study are available in the Dryad Digital Repository: https://doi.org/10.5061/dryad.zs7h44jqs
